# Nurse‐perceived facilitators and barriers to palliative care in patients with kidney disease: A European Delphi survey

**DOI:** 10.1111/jorc.12371

**Published:** 2021-03-24

**Authors:** Ilaria de Barbieri, Veronica Strini, Helen Noble, Stefano Amatori, Davide Sisti

**Affiliations:** ^1^ Department of Health Professions University Hospital of Padova Padova Italy; ^2^ Projects and Clinical Research Unit University Hospital of Padova Padova Italy; ^3^ School of Nursing and Midwifery Queens's University Belfast Belfast UK; ^4^ Department of Biomolecular Sciences, Service of Biostatistics University of Urbino Carlo Bo Urbino Italy

**Keywords:** conservative management, Delphi studies, end‐stage kidney disease, nurse, palliative care

## Abstract

**Background:**

The palliative care phenomenon is increasingly invested in all medicine and nursing fields, as care for people with kidney disease who do not wish to embark on dialysis: it encompasses a palliative approach to shared decision‐making. To deliver patient‐centred optimal care, nephrology healthcare staff should be knowledgeable about palliative care and the appropriate conservative management approach.

**Objective:**

This paper aimed to explore, using a Delphi survey, the barriers and facilitators to palliative care in patients with kidney disease.

**Design:**

An e‐Delphi technique with three questionnaire rounds was performed; statements were generated using Likert scales.

**Participants and Measurements:**

A list of 80 statements related to palliative care in patients with kidney disease was divided into facilitators and barriers. Questionnaires were administered to 13 nephrology nurse experts in some European countries.

**Results:**

Seven items were removed from the list of 80 statements after the first round of the Delphi study; eight items achieved a significant change of the mean between round two and three, whereas internal stability emerged in all the remaining items.

**Conclusions:**

Specific training and education in palliative care emerged as a facilitator, as well as the role of spiritual and beliefs and the role of family and caregiver. The main barriers were represented by the differences in cultures, beliefs, and practices and by the lack of experience in the role of the staff in palliative care. These statements provide a platform for future research to improve palliative care practice in patients with kidney disease.

## INTRODUCTION

The palliative care phenomenon is increasingly invested in all fields of medicine and nursing. In recent years, there are increasing numbers of patients with kidney failure (Lazenby et al., 2016), and with them, the possibility of choosing alternative ways of treatment to achieve the best quality of life in the last period of their life. The person with kidney disease must face the inevitable prospect of dying, and the nephrology nurse is often the first person who hears the patient's request to withdraw from dialysis (Price, 2003). Healthcare professionals regularly encounter patients considering complex treatment decisions as they approach kidney failure (Song et al., 2013). Options include peritoneal dialysis requiring support from another individual, treatment 7 days a week, a permanent catheter, risk of infection/peritonitis; haemodialysis, which usually requires attachment to a dialysis machine for up to 4 h three times a week or kidney transplantation available to those who are fit and able to withstand surgery (NICE, 2018). For some older, frailer patients with additional comorbidity and unable to tolerate transplantation, the side effects and obligations of dialysis may be something they do not wish to endure; for example, travelling to hospital three times a week for haemodialysis or carrying out multiple peritoneal dialysis bag exchanges (Brown et al., 2015; Elliott et al., 2014; Morton et al., 2012; Noble et al., 2009). In addition, dialysis may not be of benefit (Hussain et al., 2013; Verberne et al., 2016). Patients in this situation may opt for different palliative approaches, such as conservative management (CM). This type of treatment represents a holistic patient‐centred approach to care which encompasses shared decision‐making, management of kidney disease to delay progression, symptom management, comprehensive communication including advance care planning, psychological support, spiritual support, social and family support, and cultural care (Davison et al., 2015; Noble et al., 2015). CM may be one of the preferred treatments in the field of palliative care for her holistic taking charge, but it is not the only existing approach.

As reported in the literature, another approach could be RSC, which is emerging as a central topic in nephrology. It deals with a concept that is similar to palliative care (PC), end‐of‐life care, and conservative patient management (Noble et al., 2007), but with some differences. Although very similar, they are not synonymous and as such require a definition.

The concept of RSC was analysed by Noble et al. (2007), defining specific points to describe it:

- RSC must be available from the time of diagnosis until the patient's death, with an emphasis on a clear prognosis and the impact of advanced kidney disease.
- The approach to treatment must be multidisciplinary to avoid the medicalisation of the patient's psychological needs.
  In some situations, the palliative physician could only follow patients with oncological pathologies at the end of their life, not covering all the patients with nononcological pathologies.
- Patient caregiver support is essential in RSC. The pressures undergone by financial budgets are an important factor (Moss, 2000). In family kidney disease, unaffected caregivers are used to having an additional emotional burden that may be initially underestimated (Alvarez‐Ude et al., 2004).
- Communication skills ensure a correct and adequate shared decision‐making process. The goals of RSC are not achievable without training in communication skills (Holley, 2007).

To overcome the barriers that RSC can place due to this structured definition, which is missing in some European countries, and the consequent impacts both in terms of training and assistance, the research focussed on the palliative approach, which can embrace every aspect of this type of care.

## LITERATURE REVIEW

Tonkin‐Crine et al. (2015) found that staff supporting patients had a strong influence on patients with respect to their treatment and treatment options, and only a small number had been informed about expected disease progression. In a study by Bull et al. (2014), 90% of the patients interviewed indicated as fundamental, receiving detailed information on their prognosis, as well as a further 80%–85% of respondents considered it necessary to be able to choose their treatment options, including withdrawal from dialysis (Bull et al., 2014).

The literature strongly underlines the importance of involving patients and families in end‐of‐life decision making. Medical communication regarding prognosis has a huge impact on the patient's final choice. Differences in training have been identified as a factor creating variation in end‐of‐life decision making (Holley, 2007).

An example of a different approach to a palliative choice is CM: Literature suggests that some people with kidney disease are ill‐informed about CM and that their treatment decisions are poorly supported in clinical practice (Song et al., 2013; Wong et al., 2016). Nephrology CM programmes vary across the world, and few guidelines or policy directives are identifying the core components and essential practices of such programmes (Davison et al., 2015). In some countries, CM is not offered as a treatment option; in others, patients have no option but to be managed conservatively as dialysis is financially prohibitive. Nephrology staff, therefore, face challenges but also opportunities in this area (Noble et al., 2009).

Nephrologists are becoming increasingly aware that palliative care is not merely management of the illness at the end of life, but rather a supportive care pathway that leads over time to a dignified end of life for the patient (Holley, 2005).

Palliative care considers death as a natural process and allows the patients to live the last phase of their existence in the best possible way, remaining adherent to the values, beliefs, culture, and religion of the individual. It encourages patients to express their wishes on how to live the last phase of their life to give them customised support (Price, 2003; Young, 2009).

The palliative care phenomenon is increasingly investing in all fields of medicine and nursing. In recent years, patients with end‐stage kidney disease [ESKD] appear mostly elderly (>65 years), with multiple comorbidities and complex healthcare needs, and their number is growing exponentially (Lazenby et al., 2016). In the same way, the possibility of choosing alternative treatments is increasing.

Sturgill and Bear (2019) outlined the problem of unmet palliative care needs for patients with kidney failure and barriers to improving palliative care from the physician's point of view. It emerged that there are some disincentives to palliative care in the CKD population: Underdeveloped models of care for severely ill patients with CKD, misaligned incentives between dialysis and palliative care, and uneven access to speciality palliative care (this last specifically in the United States of America).

In contrast, the most developed model of outpatient palliative nephrology comes from Australia, where patients are followed by multidisciplinary teams comprising palliative care and nephrology specialists (Sturgill & Bear, 2019).

The quality of care for patients with ESKD approaching the end of life needs improvement (O'Halloran et al., 2018; Roderick et al., 2015) and conservative care programmes are emerging internationally with the goal of better addressing the end‐of‐life needs of patients with kidney disease, in particular in a palliative care vision. Little is known about the palliative care nursing practice in patients with kidney disease across the world and the views of staff caring in this context (Lazenby et al., 2016). In addition, the facilitators and barriers to palliative care have not been clearly articulated.

As regards nephrology nurses, it is relevant that there is a lack of knowledge about when and how to approach the topic with their patients (Ceccarelli et al., 2008). They need more education, practice, and mentoring to talk about end‐of‐life at the right time and in the right way (Ceccarelli et al., 2008). Price (2003) claims that is particularly important that the nephrology nurse incorporate precepts of palliative and end‐of‐life care into the Shared Decision Making Guidelines for initiation or for withdrawal from dialysis, and provides them in collaboration with the nephrologist in the care of patients.

Some nurses and social workers express frustration when a patient's wishes are ignored by physicians at the end of life (Sellars et al., 2017), whereas they feel that they have treated the dying patient with dignity and respect by honouring his wishes (Haras, 2008).

The withdrawal for dialysis treatment for people with ESKD places renal nurses in a unique situation. The withdrawal from dialysis treatment could signify the end of life, the creation of a predictable death and the end of a long‐term relationship (Johnson & Bonner, 2004).

Currently, there are no data on barriers and facilitators from the nurse's point of view in the European countries.

Furthermore, there are no studies in the literature that use the Delphi technique for the creation of a questionnaire, addressed to nurses or other health professionals, to investigate their experiences or opinions on palliative care in patients with kidney disease.

This paper aims to explore the barriers and facilitators to palliative care in patients with kidney disease, across some European countries, through a Delphi study.

## MATERIALS AND METHODS

A three‐round e‐Delphi technique was undertaken. An e‐Delphi approach is defined as the use of the modified Delphi technique via an electronic/web‐based medium (Gill et al., 2013; Keeney et al., 2006; Keeney et al., 2011). Using consecutive surveys, it is possible to collect, evaluate, and tabulate opinions of experts in the area under study. The characteristics of the Delphi technique are based on anonymity, iteration, controlled feedback, and statistical group response (Holloway & Wheeler, 2002). Controlled feedback and statistical group response take place in between rounds by informing individual experts about the opinions of the total expert group. The e‐Delphi technique is performed via email or online web surveys. Within healthcare research, the Delphi technique is often used to set priorities or to gain consensus about important issues (Haras, 2008; Ho et al., 2010). Iteration is obtained through a series of (typically) three 'rounds' of questionnaires in which each statement is measured and scored. Between rounds, the group's answers are statistically analysed to provide mean scores and standard deviations: The scores are communicated to the panel to reach consensus (Crisp et al., 1997). The three rounds were completed within 3 months, from the first of May to the first of September 2018. Three rounds were implemented to assure the internal stability between the replies: The group of experts was asked to participate in each phase of the Delphi Study until the statistical analysis was adequate to confirm each statement and to define the final questionnaire. The answers to each statement were collected through a rating on a four‐level Likert scale.

**Figure 1:**
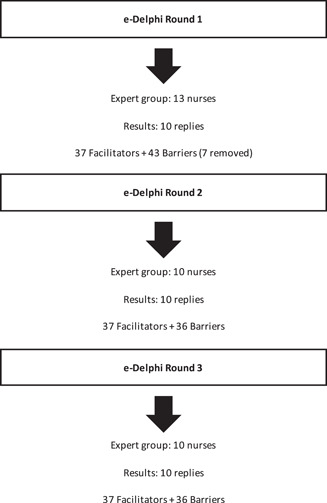
The flow chart of e‐Delphi rounds

The authors created the statements through a literature review on the topic of barriers and facilitators of palliative care in patients with kidney disease (Figure 1).

### Participants

Positive sampling was used to identify participants from three universities and five health services in ten European countries. Participants were considered experts in the area if they had postgraduate qualifications in an acute nursing area and experience in nurse education (either clinically or at a higher education institution). The inclusion criteria included: Qualified as a nurse; a member of the European Dialysis and Transplant Nurses Association/European Renal Care Association (EDTNA/ERCA); at least five years of experience in a nephrology context; a good understanding of the English language.  Experience in palliative care or conservative management was recommended but not mandatory as in many European countries (Italy, Greece, Spain, France, Germany) the advanced kidney care team does not exist formally. So, it was considered that only the more experienced nurses could have cared for people with kidney disease in a PC approach.

Nurses are recognised worldwide as the HCPs more 'bedside' and in contact with people affected by any kind of disease and their families. So, the study considered nurses as the HCP most experienced in terms of barriers and facilitators in this area, and other HCPs were not included.

There are no universally agreed on criteria for a minimum or a maximum number of experts in a Delphi Study, related to developing a critical thinking assessment tool for Australian undergraduate nurses, that of Jacob et al. (2018) included 13 nurses. Also in this study, the inclusion criteria met the requirements of 13 nurses.

Contact details for participants were obtained through the EDTNA/ERCA membership database. The 13 participants were selected based on nephrological and palliative care experience recognised within the association. Ten participants answered all rounds. Individuals were informed about the voluntary nature of the study and the need for participation in all three electronic survey rounds. The initial request to participate in the study was emailed to the 13 recognised experts, along with an explanatory statement regarding the study and a link to the survey.

Data collected were managed in agreement with General Data Protection EU Regulation (GDPR). Consent to collect and process these data was obtained from each participant, sending an email with a letter attached. Furthermore, as members of the Association, participants agreed with the Approval and Acknowledgement to Publish and store Personal Data on the Website and Database of EDTNA/ERCA due to implementation of the EU GDPR, 25 May 2018.

Briefly, each participant has given consent to the processing of data collected; the survey was conducted in an anonymous form, so it was not possible to trace each questionnaire to the specific participant. The data was stored in a specific database on a single password‐protected computer of the coauthor.

### Data analysis section

Responses to all statements were captured in a four‐level Likert scale as below:

**Table:** 

	**1**	**2**	**3**	**4**	
*Strongly disagree*	○	○	○	○	*Strongly agree*

To avoid the possibility of choosing the central value (corresponding to a neutral opinion, known as “median effect”), an even number of levels was considered; so, respondents' judgement has been forced. Rounds were analysed according to Holloway and Wheeler (2002); the paired *t* test was also applied as described by Tume et al. (2014) to calculate differences between round 2 and round 3 (significance level set at ≤0.05). The importance of the statements was determined by the highest mean and smallest *SD*.

All statements showing an average equal to or greater than 3 were considered as validated items at the end of the study. Mean quantitative analysis of the Delphi study included calculations of the mean (*SD*) and Quadratic Weighted Kappa (*κ*) values to compare chance‐eliminated agreement between rounds. Strength of agreement was codified according to Altman (1991); for *κ* = 0.81–1.00 very good agreement, for *κ* = 0.61–0.80 good agreement, for *κ* = 0.41–0.60 moderate agreement, for *κ* = 0.21–0.40 fair agreement and *κ* < 0.21 poor agreement.

Data analysis was performed using IBM SPSS V.20 software and Microsoft Excel 365.

### Ethical considerations

Anonymity was maintained, and none of the participants was provided with details of the other participants in the study. Letters of invitation were sent to participants, informing them of the purpose and nature of the study, providing assurances of confidentiality and the option to withdraw from the study at any time. Before starting the study, the Ethics Committee of Urbino University (Italy) was asked for approval. An informal answer (by email) stated that for the Delphi study design, the approval by the Ethics Committee was not mandatory, the study being not ethically sensitive (no personal information was collected, and participants were not subjected to any risks). Indeed, there are no standard guidelines on registration and Ethics Committee approval for Delphi studies.

## RESULTS

Thirteen EDTNA/ERCA nursing members were approached to participate in the survey, and all nurses agreed to take part. Ten completed all three rounds (77%), whereas the other three participants never responded.

The participants were each from a different European country: Italy, Greece, Germany, Poland, United Kingdom, Spain, Lithuania, Portugal, Denmark, Belgium.

The majority of respondents were female (80%), with a mean age of 48.8 years (median = 48; 1st quartile = 41.5; 3rd quartile = 55.5). Their areas of work were: haemodialysis (60%); haemodialysis and peritoneal dialysis (20%); acute renal setting (10%) and department of renal medicine (10%). Their working organisations were: Public hospital (80%), private hospital (10%), private specialist centre (10%). Their roles were: Staff nurse (40%), clinical nurse specialist (30%), head nurse (20%), and Director of Education (10%). Their nursing degree: Bachelor Degree (20%), Master Degree (60%), Postgraduate nephrology course after bachelor degree (20%). The average length of nephrology experience was 25 years. The response rates of items completed by all participants in consecutive rounds were as follows: 93% in round 1 (68 of 73 items), 97% in round 2 (71 of 73), and 99% in round 3 (72 of 73).

The results of the study produced a questionnaire of 73 statements divided into two domains: 37 facilitators and 36 barriers. According to Holey et al. (2007), the results section summarises the Delphi in terms of how consensus and stability evolved through rounds 1 to 3 by looking at the agreement ranking, importance rankings, and *κ* values.

The results of rounds 2 and 3 are reported below (Table 1): The results of the first round are not presented as seven statements were removed from the 80 initial statements as the mean was less than 3.0. The next two rounds were necessary to measure the internal stability of the statements kept.

**Table 1:** Results of statements of rounds 2 and 3

Statements	Round 2 Mean (*SD*)	Round 3 Mean (*SD*)	Cohen's *k*	*t* (*p*)
Facilitators				
Impartial listening	3.7 (0.7)	3.5 (0.7)	0.81	1.50 (0.17)
Active listening	3.7 (0.5)	3.8 (0.4)	0.74	1.00 (0.34)
Communicating truthfully and clearly about patients' prognosis	3.4 (0.5)	3.5 (0.5)	0.80	1.00 (0.34)
Involving the family of the patient in choosing dialysis or palliative care	3.4 (0.5)	3.4 (1.0)	0.45	0.00 (1.00)
A collaborative approach between renal services in the hospital and the community	3.3 (0.5)	3.6 (0.5)	0.44	1.96 (0.08)
Support of a renal palliative care team	3.6 (0.5)	3.8 (0.4)	0.55	1.50 (0.17)
Adequate education on approaching end‐of‐life by medical staff	3.6 (0.5)	3.8 (0.4)	0.55	1.50 (0.17)
Adequate education on approaching end‐of‐life by nursing staff	3.8 (0.4)	3.8 (0.4)	1.00	0.00 (1.00)
Reassuring patients that they will not feel abandoned if they choose palliative care	3.4 (0.5)	3.9 (0.2)	0.20	2.45 (0.04)*
Presence of a multiprofessional team	3.6 (0.5)	3.6 (0.5)	1.00	0.00 (1.00)
An environment that supports innovation. research. education and dissemination of best practices	3.7 (0.5)	3.8 (0.4)	0.78	1.00 (0.34)
A focus on symptom management and psychosocial support	3.5 (0.5)	3.7 (0.5)	0.60	1.50 (0.17)
Patient able to die in a place of their choice	3.5 (0.5)	3.9 (0.2)	0.20	2.45 (0.04)*
Good management of symptoms	3.8 (0.4)	3.8 (0.4)	1.00	0.00 (1.00)
Patients talking about approaching end‐of‐life	3.4 (0.5)	3.6 (0.5)	0.62	1.50 (0.17)
Presence of a specific plan of care which includes advanced care planning	3.9 (0.2)	3.8 (0.4)	0.62	1.00 (0.34)
Treating the dying patient with dignity and respect	3.7 (0.5)	3.9 (0.2)	0.41	1.50 (0.17)
End of life care competencies for medical staff included in university curricula	3.5 (0.5)	3.5 (0.5)	1.00	0.00 (1.00)
End of life care competencies for nursing staff included in university curricula	3.7 (0.5)	3.7 (0.5)	1.00	0.00 (1.00)
Participation of family/carers in decision‐making	3.6 (0.5)	3.3 (0.5)	0.44	1.96 (0.08)
Presence of national guidelines that support clinical practice at the end of life period	3.5 (0.5)	3.7 (0.5)	0.60	1.50 (0.17)
Providing postregistration training to nephrology nurses	3.8 (0.4)	3.7 (0.5)	0.74	1.00 (0.34)
Providing a stimulating work environment with places where teams can meet, interact and reflect	3.3 (0.5)	3.8 (0.4)	0.19	3.00 (0.01)*
Collaboration with a palliative care team in the community	3.8 (0.4)	3.8 (0.4)	1.00	0.00 (1.00)
Medical staff communicating effectively	3.5 (0.5)	3.6 (0.5)	0.80	1.00 (0.34)
Nursing staff communicating effectively	3.4 (0.5)	3.8 (0.4)	0.29	2.45 (0.04)*
Medical staff have palliative care experience	3.6 (0.5)	3.6 (0.5)	0.80	1.00 (0.35)
Nursing staff have palliative care experience	3.6 (0.5)	3.6 (0.5)	1.00	0.00 (1.00)
Presence in the hospital of a positive attitude towards palliative care	3.6 (0.5)	3.8 (0.4)	0.55	1.50 (0.17)
Implementation of standard scales for symptom assessment	3.8 (0.4)	3.8 (0.4)	1.00	0.00 (1.00)
Information for the family/carers about the protection and promotion of life until death while receiving palliative care	3.5 (0.5)	3.8 (0.4)	0.40	1.96 (0.08)
Availability of psychological support in complex communication	3.7 (0.5)	3.8 (0.4)	0.74	1.00 (0.34)
Presence of standard hospital procedures for palliative care	3.7 (0.5)	3.6 (0.5)	0.78	1.00 (0.34)
Presence of a network of nursing home staff and residential care home staff	3.6 (0.5)	3.7 (0.5)	0.78	1.00 (0.34)
Identification of cultural barriers among healthcare professionals that could prevent uptake of palliative care.	3.7 (0.5)	3.9 (0.2)	0.42	1.50 (0.17)
Knowledge about the different cultural approaches to the end of life	3.7 (0.5)	3.8 (0.4)	0.74	1.00 (0.34)
Knowledge about spiritual needs at the end of life period	3.7 (0.5)	3.8 (0.4)	0.62	1.00 (0.34)
Barriers				
Lack of training and resources to conduct difficult discussions about deterioration	3.7 (0.5)	3.8 (0.4)	0.74	1.00 (0.34)
Lack of time to conduct difficult discussions about deterioration	3.3 (0.5)	3.3 (0.7)	0.68	0.00 (1.00)
Involving family/carer at the end of life decision making	3.6 (0.7)	3.0 (0.9)	0.63	3.67 (0.01)*
Lack of collaboration between nursing and medical staff	3.5 (0.5)	3.4 (0.7)	0.86	1.00 (0.34)
Refusal by the patient to accept deterioration and approaching death	3.3 (0.7)	3.4 (0.7)	0.88	1.00 (0.34)
Refusal by the family to accept deterioration and approaching death of the patient	3.6 (0.5)	3.6 (0.5)	1.00	0.00 (1.00)
Fear of staff to family reactions to palliative care	3.5 (0.5)	3.2 (0.6)	0.57	1.96 (0.08)
Feel unprepared to start difficult conversations. and having a fear of using the wrong words	3.6 (0.5)	3.4 (0.5)	0.62	1.50 (0.17)
Cultural beliefs and practices	3.4 (0.7)	3.6 (0.5)	0.72	1.50 (0.17)
Spiritual beliefs	3.3 (0.7)	3.6 (0.7)	0.68	1.96 (0.08)
A lack of knowledge about which patients will benefit from renal replacement therapy rather than palliative care	3.4 (0.5)	3.4 (0.7)	0.71	0.00 (1.00)
Individual survival and quality of life predictions difficult in the elderly with end‐stage kidney disease	3.2 (0.4)	3.4 (0.7)	0.38	1.00 (0.34)
Absence of adequate palliative care services in rural areas	3.4 (0.5)	3.6 (0.7)	0.44	1.00 (0.34)
The patient and the patient's family think that withdrawing from dialysis is the same as euthanasia	3.5 (0.5)	3.3 (0.5)	0.60	1.50 (0.17)
Nephrologists focus on biomedical factors and have an inherent instinct to prolong the life	3.5 (0.5)	3.5 (0.5)	1.00	0.00 (1.00)
Nephrologists try and maintain hope for the future	3.4 (0.5)	3.2 (0.6)	0.69	1.50 (0.17)
Regret in patient and family about stopping dialysis	3.5 (0.5)	3.2 (0.4)	0.40	1.96 (0.08)
Limited evidence to support renal palliative care in the literature	3.5 (0.5)	3.2 (0.6)	0.57	1.96 (0.08)
Family/carer's involvement in the decision‐making process	3.6 (0.7)	3.3 (0.5)	0.32	1.41 (0.19)
Clinicians influencing the patient to make a particular decision	3.8 (0.4)	3.2 (0.4)	0.12	3.67 (0.01)*
Nurses influencing the patient to make a particular decision	3.6 (0.5)	3.1 (0.6)	0.36	3.00 (0.01)*
Shared treatment decision‐making is not a common term in the renal unit	3.5 (0.7)	3.3 (0.7)	0.78	1.50 (0.17)
End‐of‐life discussions are often not started by the health care team	3.3 (0.5)	3.4 (0.5)	0.78	1.00 (0.34)
Difficulty in estimating prognosis	3.6 (0.5)	3.1 (0.7)	0.49	3.00 (0.01)*
Death considered a taboo subject	3.3 (0.7)	3.4 (0.7)	0.88	1.00 (0.34)
Nurses' lack of experience conducting palliative care	3.7 (0.5)	3.6 (0.5)	0.78	1.00 (0.34)
Medical staff lack of experience conducting palliative care	3.7 (0.5)	3.8 (0.4)	0.74	1.00 (0.34)
Beliefs in the preservation of hope and life	3.6 (0.5)	3.3 (0.7)	0.59	1.96 (0.08)
Medical staff lack experience in end of life care	3.5 (0.5)	3.7 (0.5)	0.60	1.50 (0.17)
Nurses lack experience in end of life care	3.6 (0.5)	3.3 (0.7)	0.59	1.96 (0.08)
Worries about legal consequences	3.6 (0.5)	3.5 (0.7)	0.86	1.00 (0.34)
Prolonging life viewed as more important than honouring a patient's request to forgo life‐sustaining treatment	3.6 (0.7)	3.5 (0.5)	0.69	0.00 (1.00)
The family disagrees with the patient's wishes	3.6 (0.5)	3.4 (0.5)	0.62	1.50 (0.17)
Insufficient information about palliative care in the nursing university curriculum	3.6 (0.5)	3.5 (0.5)	0.80	1.00 (0.34)
Insufficient information about palliative care during medical training	3.3 (0.5)	3.6 (0.5)	0.44	1.96 (0.08)
The stigma of palliative care in some cultures as an acceptance of death	3.6 (0.5)	3.7 (0.7)	0.55	0.56 (0.59)

* Statistically significant *p* < 0.05.

Eight items achieved a significant change of the mean between 2 and 3 rounds (greater value of '*t*'), whereas internal stability emerged in all the remaining statements.

In round 2, means (±*SD*) are located between 3.3 ± 0.5 (a collaborative approach between renal services in the hospital and the community) and 3.9 ± 0.2 (presence of a specific plan of care which includes advanced care planning) for facilitators and between 3.2 ± 0.4 (individual survival and quality of life predictions difficult in the elderly with ESKD) and 3.8 ± 0.4 (clinicians influencing the patient to make a particular decision) for barriers.

In round 3, for facilitators, the lowest mean value was 3.3 ± 0.5 (participation of family/carers in decision‐making) and the highest mean was 3.9 ± 0.2 (reassuring patients that they will not feel abandoned if they choose palliative care; patient able to die in a place of their choice; treating the dying patient with dignity and respect; identification of cultural barriers among healthcare professionals that could prevent uptake of palliative care). The means ranged between 3.0 ± 0.9 (Involving family/carer at the end‐of‐life decision making) and 3.8 ± 0.4 (Clinicians influencing the patient to make a particular decision) for barriers.

**Figure 2:**
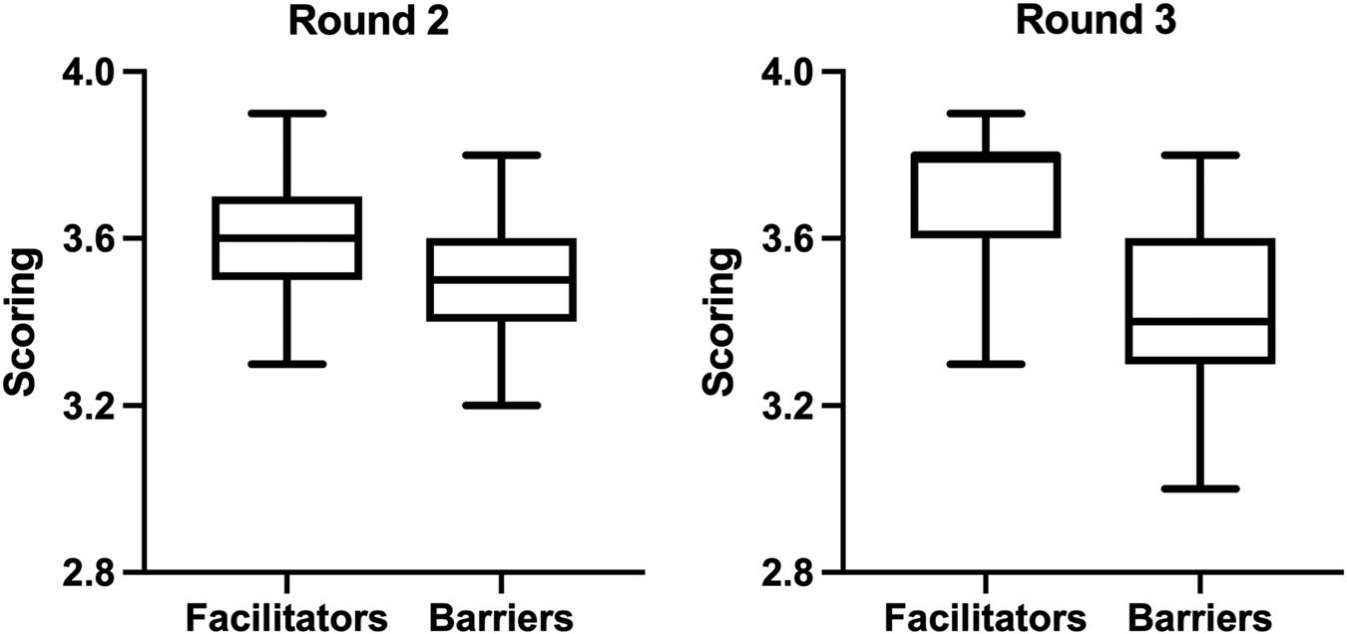
Box plots of Delphi rounds 2 and 3 mean scorings, split for facilitators and barriers. Box reports median, first and third quartile, and whiskers represent min and max scores

Considering facilitators in round 2–3 agreement, Cohen's *k* showed a good‐very good agreement (*k* > 0.61) for 24 items (59.4%), whereas only three items (8.1%) had a poor agreement (*k* < 0.21). Barriers showed a good‐very good agreement for 21 items (58.3%), whereas only one item had a poor agreement. Results for rounds 2 and 3, stratified for facilitators and barriers, are represented in Figure 2. The median value for facilitators was higher than for barriers, even if all values were higher than 3, the minimum value accepted as agreement value.

## DISCUSSION

An important facilitator appears to be the presence of a plan addressed to this specific kind of population and, as a consequence of this, the presence in the University curricula of end‐of‐life care competencies (score 3.7–3.7, that means high internal stability). Lazenby et al. (2016) affirm that all practising healthcare professionals should receive adequate preparation to support a 'good death'.

In line with this, also the items 'Adequate education on approaching end‐of‐life' by medical and nursing staff (3.6–3.8; 3.8–3.8), or 'Medical/nursing staff have palliative care competencies' have collected a good score (3.5–3.5; 3.7–3.7). Lazenby et al. (2016) again claim that a change in the culture of dialysis to ensure all the patients' information and the opportunity to discuss end‐of‐life is needed.

As regards the type of management administered to this population, the most important topic is the management of symptoms, connected with the importance of the presence of a multiprofessional team or, where it is possible, the presence of a 'Renal Palliative Care team'. Raghavan and Holley (2016) affirm that a team needs to comprise nurses, social workers, nephrologists, and a palliative care specialist who focuses on symptoms management and support the family. It is also better to initiate a multidisciplinary approach focusing on goals of care and providing palliative care to all patients (Raghavan & Holley, 2016). Advanced care planning can emphasise communication with the patient and his family (Young, 2009).

In contrast to what has just been reported, the results show that some important barriers are the 'Lack of collaboration between medical and nursing staff' (3.5–3.4) and their lack of experience conducting palliative care (nurse 3.7–3.6; medical 3.7–3.8).

As reported by Yee et al. (2011), the family plays an important role in health‐related decision making for the patient and their opinions regarding treatment plans may sometimes conflict with the patient's preferences. So, if 'Communicating truthfully and clearly about patients' prognosis' is considered a facilitator (3.4–3.5), the 'Family disagrees with the patient's wishes', 'Family/carer's involvement in the decision‐making process', with 'Cultural and spiritual beliefs' are considered barriers (3.6–3.4; 3.6–3.3; 3.4–3.6).

Death is considered a taboo subject, both from the study of Lazenby et al. (2016), and from the data emerged from this study, and the palliative care a stigma that hinders a free decision by the patients, as well as 'Regret in patient and family about stopping dialysis' (3.5–3.2).

The role of the family has a fundamental impact on the decision: The item 'Prolonging life viewed as more important than honouring a patient's request to forgo life‐sustaining treatment' has a good score (3.6–3.5), in line with the previous. The free decision of the patients is not totally free. The family could be unsure or ignorant of the progress of the disease or felt unready, unprepared or absent (Bull et al., 2014). The relationship between patients and their families creates a 'cycle of ambiguity' that confuses the end‐of‐life decision and prompts the 'executive' decision (Bull et al., 2014).

Good management of the care path is fundamental, with a good education regarding the way to make the patient and the family aware of the future, to avoid that 'The patient and the patient's family think that withdrawing from dialysis is the same as euthanasia' (3.5–3.3). Withdrawal of dialysis is legally and ethically valid: Is not considered euthanasia (Crail et al., 2013).

The role of CM in the path of the patient regards the possibility to treat the patient with dignity and respect, another item considered a facilitator. If the vision of the patient and his family is not only important but also the beliefs and values of health professionals have a clear impact on the integration of palliative care in the management of terminal patients (Fassett et al., 2011). The nephrologist nurse is often the first person who hears of the patient's request to withdraw dialysis (Haras, 2008). Nurses have to highlight the successful achievement of a good death, respecting and honouring their wishes, establishing an open dialogue with the family, continuing education programme and remaining current with published literature. No personal beliefs and values have to influence the attitude towards the patient and to influence the decision (Haras, 2008).

It is fundamental to overcome this negative view of the palliative care pathway, in line with the high score of the facilitator 'Availability of psychological support in complex communication' (3.7–3.8), to avoid the 'Lack of training and resources to conduct difficult discussions about deterioration' (3.7–3.8), considered an important facilitator too.

As Ho et al. (2010) revealed, appropriate and adequate communication is very important in end‐of‐life care. They underline how, in a country where religion is one of the more important topics, there is an influence of spiritual beliefs, and it was expected to find a connection between religion and a positive attitude to care for dying patients, whereas she found no connection between them. The principal fear of nephrologists is to be unsure about whether they are making 'the right decision or not' in these situations (Sellars et al., 2017). Sellars claims that the inability to cure is often seen as a personal failure.

Also, here the theme of education and training comes back: A good preparation towards the communication could support the nephrologist to help the patient to enact his choice in the face of conflicting opinion from colleagues or family members (Sellars et al., 2017).

Another important topic to take into consideration is the role of spiritual beliefs: The topics 'Identification of cultural barriers among healthcare professionals that could prevent uptake of palliative care' (3.7–3.9), 'Knowledge about the different cultural approaches to the end of life' (3.7–3.8) and 'Knowledge about spiritual needs at the end of life period' (3.7–3.8) are considered facilitators, whereas 'Cultural beliefs and practices' (3.4–3.6) and 'Spiritual beliefs' (3.3–3.6), 'Nephrologists try and maintain hope for the future' (3.4–3.2) and 'Beliefs in the preservation of hope and life' (3.6–3.3) barriers. Renal nurses have significant knowledge and experience in the management of renal failure. Still, they lack the necessary skills to assess and explore the physical, psychosocial and spiritual concerns of the patients and their families in this phase (Johnson & Bonner, 2004).

## LIMITATIONS

The limitations include the fact that the EDTNA/ERCA's members selected do not represent all the European countries, they are members of an association specialising in nephrology and dialysis, some of them are managers and no longer address the issue of direct assistance, so, therefore, the results could be biased in this study. In the study we involved only nurses and not other HCPs: In further studies, the points of view of medical and allied staff could be considered as well.

Due to the small sample size of experts in each country, no attempt has been made to compare priority research topics between countries.

Limitations also include those correlated with the Delphi technique: surveys are usually slow and time‐consuming and lack the stimulation provided by face‐to‐face meetings (Enzer et al., 1971) and, therefore, can contribute to the dropping out of panel members.

## CONCLUSION

Experts nominated by EDTNA/ERCA Association rated 80 statements through a Likert Scale, according to the Delphi technique.

The final questionnaire included 73 statements divided into two domains: 37 facilitators and 36 barriers. The fundamental points emerged from the analysis of the Likert scale scores, these were considered the main ones in the questionnaire, thanks to the high scores obtained. These are the promotion of the creation of nephrological palliative care teams and support for communication between different professionals; promotion of a common background and training in palliative care beyond the university path for each professional, to support shared and conscious decision‐making of the patient; the implementation of compassionate, ethical and effective palliative care plans that could offer hope for an improved quality of life and death for these patients and healthcare professionals.

## IMPLICATIONS FOR CLINICAL PRACTICE

Further nursing research is needed to develop tools or plans to evaluate a good palliative care pathway. Due to the limitations of this study, to have more information about palliative care, it is recommended to have a more detailed study, which encompasses all the European countries and nurses with different training and experience on the topic. This questionnaire can be the starting point for investigating this phenomenon at a wider European or international level. These results can be the first to guide the clinical practice of professionals, the role of university and field training in the palliative approach and evaluate the differences between different cultures/religions/areas and the orientation of professionals.

## CONFLICT OF INTERESTS

The authors declared that there are no conflict of interests.

## AUTHOR CONTRIBUTIONS

Ilaria de Barbieri is the principal project leader who conceived the study, participated in design and coordination, read and approved the final manuscript. Veronica Strini and Helen Noble participated in design and coordination, undertook interviews and helped to draught manuscript, read and approved the final manuscript. Stefano Amatori and Davide Sisti analysed the data, helped to draught the manuscript and approved the final manuscript.
